# A case of recurring perioperative circulatory arrest: mind the autonomic nervous system

**DOI:** 10.1007/s10286-023-00953-x

**Published:** 2023-06-07

**Authors:** Ulrich Limper, Dorothee Keipke, Lars Lindenbeck, Friederike Lanz, Claudia Kramer, Axel Meissner, Frank Wappler, Thorsten Annecke

**Affiliations:** 1 Department of Anaesthesiology and Critical Care Medicine, Merheim Medical Center, Witten/Herdecke University, Ostmerheimer Straße 200, 51109 Cologne, Germany; 2 German Aerospace Center (DLR), Institute of Aerospace Medicine, Cologne, Germany; 3 Department of Cardiology, Merheim Medical Center, Cologne, Germany

**Keywords:** Autonomic nervous system, Perioperative, Anaesthesia, Cardiopulmonary resuscitation, Right bundle branch block, Vagus nerve, Sympathicus

## Abstract

We report the case of an elderly woman who developed recurring episodes of unexplained cardiocirculatory arrest. The index event appeared during surgery to fix a fracture of the ankle and consisted of bradypnea, hypotension and asystole, coherent with a Bezold–Jarisch-like cardioprotective reflex. Classical signs of acute myocardial infarction were absent. Yet, occlusion of the right coronary artery (RCA) was observed and successfully revascularized, whereupon circulatory arrests vanished. We discuss several differential diagnoses. Unexplainable circulatory failure, with sinus bradycardia and arterial hypotension, despite lack of ECG signs of ischemia or significant troponin levels, suggest the action of cardioprotective reflexes of the autonomic nervous system. Coronary artery disease is a common source. Attention to cardioprotective reflexes should be taken in the case of unexplained cardiac arrest without overt reasons. We recommend performing coronary angiography to exclude significant coronary stenosis.

Editor,

Reflexes of the autonomic nervous system prevent the myocardium from irreversible damage in case of ischaemic stress. However, in the case of hyperreactive reflexes, cardiocirculatory arrest may occur. Therefore, albeit uncommon, autonomic reflexes should be considered in the case of sudden cardiac arrest without any overt cause.

## Case description

An 80-year-old woman fractured her left ankle (Weber type C) after a fall at home. She had a past medical history of peripheral arterial disease, smoking, relapsed multiple myeloma treated with carfilzomib and dexamethasone, chemotherapy-associated chronic kidney failure, pancytopenia, hypertension and heart failure with preserved ejection fraction and diastolic dysfunction I-II° (NT-proBNP values 1400 pg/mL and higher). Revascularization of the proximal right coronary artery (RCA) had been successfully performed 3 years earlier with implantation of a drug-eluting stent. She had impaired physical capacity of 2–3 metabolic equivalents and heart failure classified as NYHA III°. Two weeks earlier, a 24-h ECG had shown no relevant arrhythmias. The patient was on regular antihypertensive (amlodipine, candesartan, bisoprolol), loop diuretic (torasemide), lipid-lowering (rosuvastatin) and antiplatelet (ASA) treatment. On examination, she was overweight (body mass index [BMI] 35 kg/m^2^) and had an episode of arterial hypertension but without chest pain or dyspnoea and was feeling fit for surgery. A blood test confirmed pancytopenia and chronic kidney failure.

The patient was referred to emergency surgery for external fixation of the fracture under distal sciatic nerve regional anaesthesia and sedation. After an uneventful distal sciatic nerve block under ultrasound guidance (250 mg mepivacaine and 150 mg ropivacaine), continuous sedation was started (2 mg/kg/h propofol, 0.05 µg/kg/min remifentanil).

**Fig. 1:**
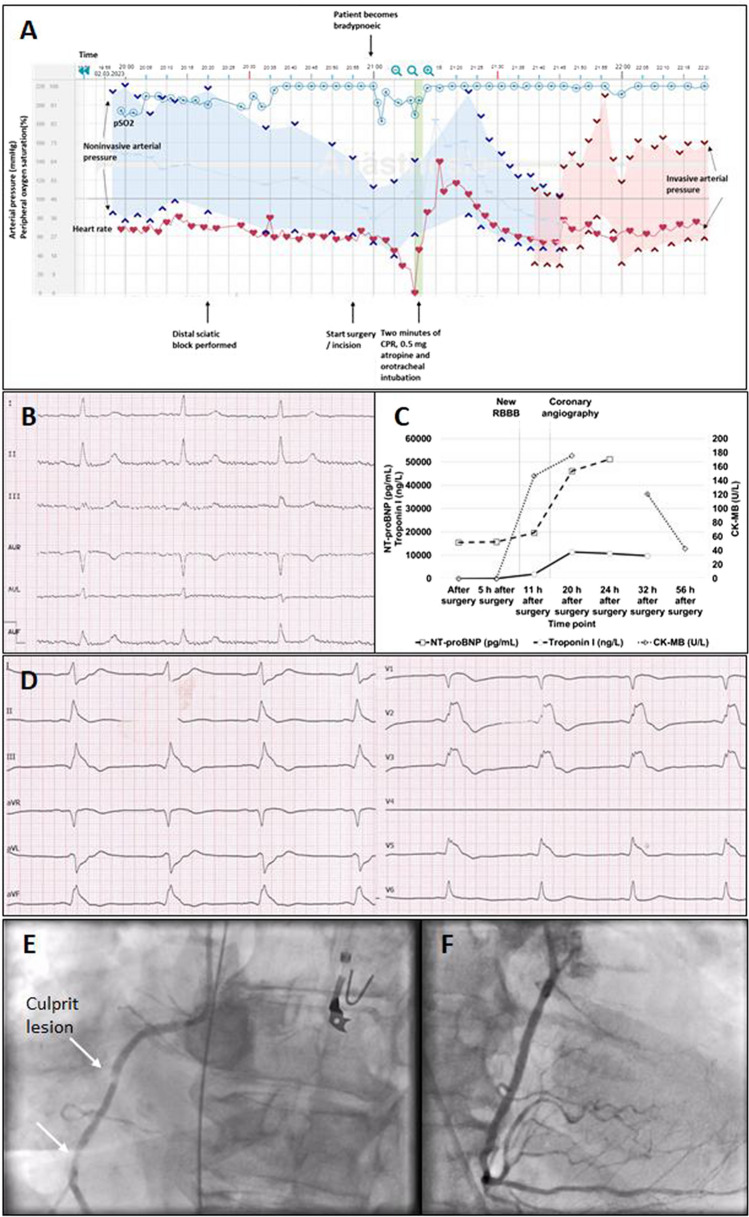
**A** Digital anaesthesia record. The hypertensive patient receives a single shot of distal sciatic nerve anaesthesia and light sedation with propofol and remifentanil. Shortly after start of surgery, the patient develops bradypnea and mild hypoxemia, and heart rate starts to decline in parallel to blood pressure, suggesting an autonomic reflex mechanism. Although sedation is stopped, cardiodepression continues, resulting in asystole. **B** 12-lead resting ECG readings at hospital admission showing normofrequent sinus rhythm without signs of ischemia. **C** Perioperative evolution of cardiac biomarkers. Despite repeated episodes of cardiopulmonary resuscitation, the increase of CK-MB and hs troponin I are delayed. The occurrence of a new right bundle branch block (RBBB) precedes the increase of CK-MB and hs troponin I. **D** New, malignant right bundle branch block. **E** Angiography of the right coronary artery showing two stenoses in the descending part (white arrows) with the proximal lesion as the likely culprit lesion. **F** Coronary angiography of the right coronary artery after revascularization

Approximately 50 min later the patient developed hypotension and bradycardia followed by asystole (Fig. 1 A). Atropine 0.5 mg and chest compression for 2 minutes resulted in recurrence of spontaneous circulation. Thereafter surgery was finished uneventfully under general anaesthesia. A post-resuscitation computed tomography (CT) scan did not reveal relevant findings. The patient was transferred, sedated and mechanically ventilated with mild vasopressor support (norepinephrine 0.01/µg/kg/min) and without malignant arrhythmias, to the intensive care unit. However, during the subsequent 8 hours, she developed 15 episodes of hypotension and unexplained cardiac arrest, with the longest episode lasting 40 min. Lowest and highest systolic and diastolic arterial pressure readings were 51/29 mmHg and 276/88 mmHg, respectively. We assumed local anaesthetic toxicity causing cardiac arrest; however, lipid resuscitation remained without success. Other reversible causes (Hs and Ts) were regularly excluded. At that time, blood gases were unremarkable. Trans-oesophageal echocardiography did not reveal any severe valvular condition or wall motion abnormality. Trans-thoracic echocardiography excluded profoundly reduced left ventricular function. CK-MB and high-sensitivity (hs) troponin remained low (Fig. 1 C), and 12-lead ECG was without classical signs of ischemia (Fig. 1 B). Yet, we found a new right bundle branch block (RBBB) (Fig. 1 D). The patient required vasopressor (norepinephrine 0.3 µg/kg/min) and inotropic (epinephrine 0.1 µg/kg/min) support to achieve haemodynamic stability between the arrest intervals. Vasopressin (3 I.E./h) was added later. Heart rate remained rather low (45/min). A mechanical reanimation device was installed for intermittent CPR and an external pacer for temporary stimulation. Despite successful external pacing, subsequent episodes of critical hypotension with functional cardiac arrest could not be avoided. Transpulmonary thermodilution confirmed preserved cardiac function under inotropic therapy (cardiac index 2.59 L/min/m^2^, cardiac function index 5.2 min^−1^, stroke volume 69 mL). Later the patient developed severe lactic acidosis (11.5 mmol/L, pH 7.02) which we treated with dialysis.

Eleven hours after surgery and after recurring episodes of prolonged cardiopulmonary resuscitation CK-MB and hs troponin I started to rise (Fig. 1 C). In this situation, we re-classified the RBBB as relevant and performed coronary angiography which revealed chronic high-grade right coronary ostial occlusion (in-stent thrombosis, lumen 3.0 mm^2^) and high-grade stenoses in the proximal and the descending parts of the RCA (Fig. 1 E). A stent was successfully placed (Fig. 1 F). The left coronary artery was unremarkable. After revascularization, no further episode of cardiac arrest appeared, RBBB vanished and heart rate accelerated again. Fortunately, despite prolonged and recurrent episodes of cardiac arrest, the patient developed no neurological sequelae.

## Discussion

We report the case of an 80-year-old woman who developed recurring episodes of unexplained functional cardiocirculatory arrest. We considered several differential diagnoses.

### Systemic local anaesthetic toxicity (LAST)?

The appearance within the first hour of anaesthesia was suggestive because approximately 70% of the events appear in the first hour, and 24% have an isolated cardiovascular finding [8]. Cardiac disease, especially reduced contractility or ischaemia, increases the risk for LAST. However, we did not recognize serious arrythmias, and lipid rescue failed to solve the situation. Plasma levels of mepivacaine (measured: 1.3 mg/L; toxic dose: 5–6 mg/L) and ropivacaine (measured: 0.21 mg/L; toxic dose: 0.48 mg/L) [5] immediately after surgery were below toxic thresholds; therefore, we finally classified LAST to be unlikely.

### Propofol infusion syndrome?

A Brugada syndrome-like ECG pattern and cardiac failure is typical. It is dose-related and usually occurs after long-term sedation (> 48 h) at high doses (> 4 mg/kg/hr), making this differential diagnosis very unlikely in this case [1]. However, propofol was stopped immediately in the ICU because of its negative inotropic effects.

### Perioperative myocardial infarction?

Myocardial infarction is subclassified into the classical type 1 which is caused by plaque rupture occlusion of cardiac coronary vessels and the non-obstructive type 2 which is caused by a prolonged mismatch between cardiac oxygen demand and oxygen supply [4]. The greater the stenosis of the coronary vessel is, the more likely type 2 develops and the less likely type 1 [6]. New left bundle branch block or ST elevations are ischaemia markers; however, RBBB can obscure these signs [11, 15]. Proximal occlusion of the RCA predisposes to bradycardia [13]. Myocardial enzymes in blood indicate tissue necrosis. Dyspnoea and chest pain are classical symptoms, which may be obscured by anaesthesia. However, ECG, echocardiography and myocardial enzymes remain meaningful. Our patient, although having profound stenosis of the RCA, never complained about dyspnoea or chest pain, myocardial enzymes remained ambiguous, ECG lacked clear signs of ischaemia, and echocardiography showed no significant wall-motion abnormalities. Only after hours, our patient progressed into type 2 myocardial infarction.

### Stress cardiomyopathy Takotsubo syndrome?

Sympathetic nerval storm or a flood of endogenous catecholamines can provoke stress cardiomyopathies, for instance Takotsubo syndrome. Several of its clinical features applied to our case [12]. Women account for 90% of all the Takotsubo syndrome cases. Acute physical or mental stress precedes the syndrome, and cancer is a risk factor. In contrast to acute myocardial infarction, patients with Takotsubo syndrome exhibit only minor increases in troponin and CK-MB but high levels of NT-proBNP (Fig. 4). On the other hand, typical wall-motion abnormalities, profoundly reduced ventricular function and ischaemic aberrations on ECG were absent in our case. Hence, we excluded stress cardiomyopathy as the major problem of our patient.

### Chemotherapy cardiotoxicity?

The patient was treated for multiple myeloma with the protease inhibitor carfilzomib and with dexamethasone and had received the last cycle the day before hospital admission. Protease inhibitors have an increased risk for serious cardiovascular events such as heart failure with preserved ejection fraction and acute coronary syndrome [7]. Measurement of natriuretic peptides, which were chronically elevated in our patient, are recommended for cardiac risk stratification protease inhibitor therapy [2]. The patient’s chemotherapy had reduced her cardiac function and increased her perioperative risk.

### Autonomic reflex?

The initial intraoperative bradycardic event of our patient reminded us of cardioinhibitory syncope (Fig. 1A) and let us consider cardioinhibitory reflexes of the autonomic nervous system as the starting point of the incident. The neural control of circulatory function is complex, including pressure and chemoreceptors in the different heart chambers, the coronary arteries and the great vessels of the thorax [10]. Cardioprotective reflexes arise from the activation of cardiac inhibitory sensors by ischaemia of the inferior-posterior wall of the left ventricle and are transmitted via vagal afferents. The right coronary artery is usually the supplying vessel of this ventricular area. Stimulation of this reflex, for instance by occlusion of the right coronary artery, increases parasympathetic activity and inhibits sympathetic activity, resulting in bradycardia and hypotension [9]. These responses have been attributed to the Bezold–Jarisch reflex and have been repeatedly associated with a failing cardiovascular system during myocardial ischaemia and infarction and non-invasive and invasive cardiological testing [3]. Interestingly, B-type natriuretic peptide enhances the Bezold–Jarisch reflex [14], which may have caused enhanced reflexes in our patient. As final explanation of our case, we supposed that the patient had a chronic failing heart because of her long-lasting cardiotoxic chemotherapy and a stenosis of the right coronary artery. When this heart became ischaemic by perioperative stress, cardioinhibitory reflexes caused recurring circulatory depressions which finally resulted in type 2 myocardial infarction. Coronary revascularization solved the problem.

## Conclusion

Unexplained cardiac arrest with manifest recurrent episodes of sinus bradycardia and hypotension and exclusion of overt reasons suggests activation of autonomic cardioprotective reflexes. Coronary artery disease is a common source. However, there is still no scientific evidence that individuals with these autonomic reflexes should undergo coronary angiography on a routine basis.

## Data Availability

All data supporting the findigs of this case report are available within the paper.
